# Oral and Gut Microbiota Dysbiosis Due to Periodontitis: Systemic Implications and Links to Gastrointestinal Cancer: A Narrative Review

**DOI:** 10.3390/medicina60091416

**Published:** 2024-08-29

**Authors:** Yaman Sulaiman, Ingrida Marija Pacauskienė, Renata Šadzevičienė, Rugile Anuzyte

**Affiliations:** Clinic of Dental and Oral Pathology, Faculty of Odontology, Medical Academy, Lithuanian University of Health Sciences, Eivenių Str. 2, LT-50161 Kaunas, Lithuaniaingridamarija.pacauskiene@lsmu.lt (I.M.P.); rugile.anuzyte@stud.lsmu.lt (R.A.)

**Keywords:** oral microbiota, gut microbiota, dysbiosis, periodontitis, systemic diseases, gastrointestinal cancer

## Abstract

Periodontitis can disrupt oral and gut microbiota, leading to dysbiosis that affects overall systemic health. Besides the spread of periodontal pathogens by the hematogenous route, they can also be translocated into the gastrointestinal tract, possibly intervening in the neoplastic process in the gastrointestinal tract. This manuscript reviews the relationship between oral and gut microbiota due to periodontitis, discussing systemic health implications and potential links to gastrointestinal cancer. This article highlights the significance and effect of dysbiosis in the gut, emphasizing the importance of maintaining oral health to prevent systemic diseases. Lastly, it will go through therapeutic innovations such as probiotics and oral microbiota analysis tools for systemic disease detection. These findings will mark the integration of oral health management in clinical practice to lower systemic disease risk and improve overall patient outcomes. *Aim of work*: This manuscript aims to unravel the pathological interaction between oral and gut microbiota and their bidirectional effect on systemic diseases. *Materials and methods*: The review was performed using the MEDLINE and ScienceDirect databases. Reviewed articles were published in English between the year 2015 and 2024. The search used keywords such as (“oral microbiota” AND “periodontal disease”) OR (“oral microbiota” AND “gastrointestinal cancer”) OR (“Porphyromonas gingivalis” AND “periodontal disease”) OR (“Helicobacter pylori” AND “gastric cancer”) OR (“gut microbiome” AND “inflammatory bowel disease”) OR (“oral microbiome” AND “systemic diseases”). *Conclusions*: The dysbiotic change in the oral cavity due to periodontitis is linked directly and indirectly to systemic diseases such as IBS, neurodegenerative diseases, muscle joint diseases, respiratory infections, and gastrointestinal cancer; this underscores the importance of maintaining oral hygiene for prophylaxis of oral diseases and the prevention of systemic diseases. A better understanding of the interconnections between oral health and systemic diseases will integrate oral health management to offer new prevention, diagnostic, and treatment opportunities to improve overall patient outcomes.

## 1. Introduction

The oral cavity and the digestive system host the most diverse and complex microbial organisms in the human body. Microbial organisms collectively form microbiota, including bacteria, fungi, archaea, protists, helminths, and viruses [1]. The microbiota in the oral cavity plays a significant role in maintaining oral homeostasis by stimulating salivary antimicrobial agents that suppress the spread of pathogenic bacteria [2]. While gut microbiota plays a similar role to oral microbiota, they are also involved in colonization resistance, intestinal barrier protection, metabolic functions, and the development of immune cells [1].

The diversity of oral microflora becomes disturbed due to diseases and many lifestyle-associated factors, such as smoking, poor oral hygiene, and periodontal disease. Dysbiosis worsens with the spread of periodontal bacteria that enter and flourish in the body [3,4]. These periodontal bacteria have been linked to the development of various systemic diseases. The pathogenesis and etiology of these diseases show an interplay of oral and gut microbiota [5].

The present article investigates the impact of dysbiosis between oral and gut microbiota on the development of systemic disease and gastrointestinal cancer. It focuses on the pathogenesis and alteration of oral and gut bacteria due to periodontitis while discussing current therapeutic innovations. A better understanding of the relationship between oral health and systemic diseases will integrate oral health management to offer new prevention, diagnostic, and treatment opportunities to improve overall patient outcomes. 

## 2. Materials and Methods

The PubMed and ScienceDirect databases were used to perform the literature search, which included many documents related to health and disease. The search used keywords such as (“gut microbiota” AND “periodontal disease”) OR (“oral microbiota” AND “gastrointestinal cancer”) OR (“Porphyromonas gingivalis” AND “periodontal disease”) OR (“Oral *H. pylori*” AND “gastric infection”) OR (“gut microbiome” AND “inflammatory bowel disease”) OR (“oral microbiome” AND “systemic diseases”).

The search was filtered to include “Review”, “Systemic review”, and “Clinical trials” paper types published in English within the past 10 years. The obtained documents were thoroughly reviewed to gather more interesting and relevant references. Information was collected from a narrative review on oral and gut bacteria topics, including their role in nutrition, antibacterial capacity, metabolism, dysbiosis, the immune system, inflammatory diseases, interlink, and the bidirectional connection of oral and gut bacteria.

**Figure 1:**
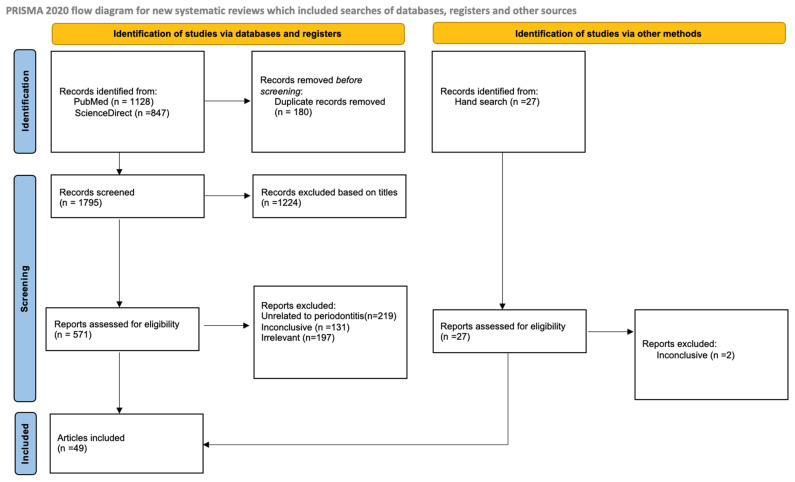
PRISMA flow diagram.

The article review and data extraction processes were performed according to the PRISMA flow diagram (Figure 1). The initial electronic search found a total of 1975 articles, 180 of which were eliminated as duplicates. After titles and abstracts were reviewed, an additional 1224 articles were filtered as not relevant. A total of 571 reports were assessed for eligibility; of these, 219 were excluded as they were unrelated to periodontitis, 131 were excluded as they were inconclusive, and 197 were excluded as they were irrelevant. A total of 27 other articles were identified through hand search and assessed for eligibility, with 2 being excluded as inconclusive. Finally, 49 articles were included in this review.

In this narrative review, we explored various studies examining the dysbiotic interaction between oral and gut microbiota in periodontitis and its implication on systemic health and the gastrointestinal tract. A formal quality assessment tool was not used in this review; therefore, the reviews and clinical trials that were mentioned were related to the research topic and provided sufficient analyses. We are aware that a narrative review can be subject to selection bias; due to this, we made a conscious effort to include studies that reflect a wide range of perspectives and outcomes to ensure a comprehensive discussion.

## 3. Oral Microbiota

### Functions and Role

The oral cavity hosts over 700 species of bacteria that have viral role in upholding both oral and overall systemic well-being and health. The oral microbiome is the second most diverse microbial community after the gut bacteria [6]. The oral cavity makes a good environment for bacteria, providing suitable conditions and persistent nutrition. These 700 bacteria species belong to 185 genera and 12 phyla. About 32% of these species are uncultivated and 14% are unnamed. The 12 phylum that harbor oral cavity are *Firmicutes*, *Chlamydiae*, *Proteobacteria*, *Actinobacteria*, *Bacteroidetes*, *Fusobacteria*, *Chloroflexi*, *Spirochaetes*, *SR1*, *Synergistetes*, *Saccharibacteria* (*TM7*), and *Gracilibacteria* (*GN02*). 

The main bacterial genera out of the 185 which functions in the oral cavity are summarized below [7]:
Gram-PositiveGram-NegativeCocci—*Abiotrophia*, *Peptostreptococcus*, *Streptococcus*, *Stomatococcus.*Rods—*Actinomyces*, *Bifidobacterium*, *Corynebacterium*, *Eubacterium*, *Lactobacillus*, *Propionibacterium*, *Pseudoramibacter*, *Rothia.*Cocci—*Moraxella*, *Neisseria*, *Veillonella.*Rods—*Campylobacter*, *Capnocytophaga*, *Desulfobacter*, *Desulfovibrio*, *Eikenella*, *Fusobacterium*, *Hemophilus*, *Leptotrichia*, *Prevotella*, *Selemonas*, *Simonsiella*, *Treponema*, *Wolinella.*

A biofilm forms around the supragingival area of the tooth compromising 100 different species. The first colonizers are oral *streptococci,* mainly *Streptococcus mitis*. It is followed by Actinomyces and a mix of Gram-positive and -negative rods, which play a role in the formation of mature dental biofilm. When a mature biofilm is formed at the supragingival area, gingivitis occurs, which causes swelling, bleeding, and an accumulation of gingival crevicular fluid. The subgingival biofilm forms when the supragingival bacteria are left undisturbed, forming a periodontal pocket; at this stage, we can say we have entered chronic periodontitis. The subgingival biofilm consists more of Gram-negative rods such *as Porphyromonas gingivalis, Prevotella* species, *Fuscobacterium,* and *Spirochetes*, which thrive in anaerobic environments [8].

Recent studies have shown that saliva contains different varieties of periodontal pathogenic bacteria, which can also be used to determine the progression of periodontitis. *P.gingivalis* is a periodontal pathogen that is found in the subgingival sulcus in patients with periodontitis grade C. These bacteria have also been found abundantly in the saliva of periodontitis patients compared to saliva samples from periodontally healthy patients [9]. A Swedish study was conducted in which saliva samples from patients with periodontitis were taken and compared with healthy samples. An increased amount of pathogenic periodontic bacteria, i.e., *P.gingivalis*, *Filifactor alocis, Parvimonas micra*, and, especially, *Tannerella forsythia*, were found. This shows a shift in bacteria species in the oral cavity due to periodontitis [10,11].

The oral cavity serves as the main interlink between the human body and the external world. It plays a role in the homeostasis of the oral microbial environment by suppressing pathogenic bacteria and protecting us from various diseases. Periodontitis can interfere with oral microbiota by affecting its composition, causing dysbiosis. On the other hand, dysbiosis can also cause oral infectious diseases, apical periodontitis, dental caries, bone osteomyelitis, and pericoronitis [12].

The pathogenic bacteria reach various parts of the body since they enter the body systemically; that is why periodontitis has been linked to diabetes, atherosclerosis, rheumatoid arthritis (RA), and inflammatory bowel disease (IBD) [13]. It is suggested that oral microbiota can reach and alter the gut microbiota by hematogenous or eternal route (gastrointestinal tract) which causes ectopic colonization of the gut, causing the destruction of the intestinal barrier and disturbing ecological balance; this leads to intestinal inflammation [14]. A recent study has shown that oral microbiota cause the dysbiosis of gut microbiota, which leads to a decrease in alpha diversity in gut microbiota due to the ectopic colonization of periodontal pathogens. When oral and gut microbiota were compared in patients with IBD and periodontitis, it was concluded that they are nearly identical, which promotes the idea of gut ectopic colonization by oral bacteria [13].

## 4. Gut Microbiota 

### Composition and Function

Gut microbiota is the most diverse of all microbiotas in the body. Different bacterial species inhabit different parts of the intestine; for example, *Proteobacteria* and *enterobacteriaceae* are found in the small intestine but not in the colon. The diversity changes in infants until the age of 3 years; then, the diversity is almost like that of adults until the age of 70 years, and decreases thereafter. The gut microbiota diversity can change depending on many factors such as obesity, drugs, diet, hormones, and genetics. Healthy gut bacteria consist of six major phyla: *Firmicutes*, *Bacteroidetes*, *Actinobacteria*, *Proteobacteria*, *Fusobacteria*, and *verrucomicrobia*. Here, *Firmicutes* and *Bacteroidetes* predominate [14].

Gut microbiota form a symbiotic relationship with the gut mucosa, which function in many capacities, such as those related to metabolism, colonization resistance, intestinal barrier protection, and immune function regulation [15]. Gut microbiota participate in vitamin K and its precursors’ synthesis; drug metabolism is performed and xenobiotics are digested. Additionally, gut microbiota impact immune responses, mucosal plasma cells, innate lymphoid cells (ILCs), and resident macrophages. There is also an interplay of gut microbiota and the immune system, where they influence the secretion of intestinal alkaline phosphatase and IL-1 receptor-related kinase (IRAK-1), which modulate intestinal immune cells [16].

Dysbiosis in the gut can be induced by infection, prolonged inflammation, xenobiotic causes (antibiotics, drugs, food additives, and sweeteners), lifestyle, and, most importantly, the environment (high/low sugar diets). Studies have shown that gut microbiota can be altered by slight changes to the diet. A sugar-rich diet can disrupt the intestinal barrier, which will cause inflammation that will disorder the metabolism, while sweeteners induce dysbiosis by causing an overgrowth of proteobacteria [17]. 

Periodontitis induces dysbiosis in the gut microbiota, according to a study in which gut microbiomes were characterized by stool samples. The study showed a lower alpha diversity of the microbiome, where *Firmucites/Bacteroidetes* were decreased in ratio due to a loss in the amount of *Bacteroidetes*. At the same time, other phylum increased in abundance, such as *Proteobacteria*, *verrucomicrobia*, and *euryarchaeota*. Most importantly, large amounts of oral periodontic bacteria were present, including *Fusobacterium*, *Parvimonas*, *Tannerella*, *Dialister*, *Filifactor*, *Treponema*, *Eubacterium*, and especially *Prevotella* and *Porphyromonas*, which were detected in the stool samples of all groups [18].

Gut microbiota have been linked to various types of diseases; whether they are the direct cause or whether they induce changes systemically in metabolic and immune systems remains unclear [17]. A recent study has demonstrated that inflammatory diseases such as Type II diabetes, IBD, non-alcoholic fatty liver disease (NAFLD), and RA are triggered by microbiota dysbiosis in the gut [15]. The main reason is that chronic dysbiosis disrupts the functions of the intestinal barrier, leading to its destruction. Invasion by different pathogens causes the secretion of proinflammatory factors through immune system activation, which develops into inflammatory disease. Later on, toxins, bacteria, and proinflammatory factors spread through the circulatory system, leading to inflammation in various parts of the body [13]. Endotoxins such as LPS, secreted from Gram-negative bacteria, can enter the circulatory system from a destroyed intestinal barrier and trigger autoimmune diseases. LPS activates immune systems by binding to Toll-like receptor 4 (TLR4) on immune cells, which causes the production of various cytokines. LPS also induces autoantibodies that mimic host antigens, leading to an autoimmune reaction [19].

IBD is a good example of gut dysbiosis demonstrating clearly that a decrease in beneficial/commensal bacteria (*Firmicutes*, *Actinobacteria*, *and Bacteroides*) and an increase in the number of pathogenic bacteria, such as proteobacteria, result in immune activity causing a mass secretion of proinflammatory factors, such as IFN-γ and TNF-α. IFN-γ and TNF-α increase the permeability and destruction of the intestinal barrier opening the gate to the circulatory system for various toxins, bacteria, and proinflammatory factors [15,20]. 

## 5. Interactions between Oral and Gut Microbiota 

### 5.1. Swallowing and Microbial Transfer 

Around 1–1.5 L of saliva are produced daily, entering the oral cavity. Saliva contains various types of proteins, salts, calcium, enzymes, and, most importantly, bacteria. Patients with severe periodontitis have abundant amounts of bacteria such as *P. gingivalis*. It has been stated that approximately 10^8^–10^10^ *P. gingivalis* cells are ingested daily [3]. These bacteria exist in very low pH environments, allowing them to survive through bile and gastric acids until they reach the intestines. Additionally, *Actinobacillus actinomycetemcomitans*, an aggressive type of bacteria that causes periodontitis, has been found in the gut [3,19]. In addition to *Porphyromonas endodontalis*, *Campylobacter rectus*, *Dialister invisus*, *Parvimonas micra*, *Filifactor alocis*, *Slackia exigua*, *Treponema*, *Prevotella*, *oribacterium*, *Tannerella*, *Leptotrichia*, *Selenomonas*, and *Fusobacterium* have all been detected in the guts of patients with varying periodontal statuses [18]. These bacteria prove that there is an ectopic colonization of the gut by pathogenic oral bacteria. When oral bacteria flourish in the intestines, their presence causes the intestine to react excessively, causing intestinal inflammation. Good dental hygiene can reduce the risk of intestinal disease [21].

### 5.2. Mendelian Randomization Test

Since the relationship between oral and gut bacteria is bidirectional, numerous studies have tried to prove that gut bacteria impact oral health. Inflammation in the oral cavity can affect the intestine, but the issue of how gut microbiota can induce and impact periodontitis in the oral cavity is unknown. Using the Mendelian randomization test, 211 gut microbiota were studied, including 9 phyla, 16 classes, 20 orders, 35 families, and 131 genera; the results showed that 16 types of bacteria were related to periodontitis and tooth loss, and of these, 5 such *Lactobacillaceae* were associated with an increased risk of periodontitis [22]. The mechanism of how gut bacteria interfere in the pathogenesis of periodontitis is currently being studied. The main impetus behind this research is a study in which mice with colonized gut bacteria were found to have alveolar bone resorption. This suggests that gut microbiota had influenced bone metabolism, affecting the alveolar bone [3].

### 5.3. Complications and Relation with Other Systemic Diseases 

Periodontitis, which is a chronic inflammatory disease of the periodontal tissue, has a major impact on systemic health. Periodontal pathogen can either spread through a hematogenous route (circulatory blood system) or an external route (swallowing) [23]. The spreading of various pathogenic bacteria, such as *P. gingivalis* in the study of mice, via the gut has been proven to intervene in the pathogenesis of atherosclerosis, RA, diabetes, NAFLD, neurodegenerative disease, IBD, and colon cancer [13]. 

The effect of *P. gingivalis* on the gut has been proven to have an interplay in the pathogenesis of colon cancer by stimulating a proinflammatory response; tissue differentiation occurs, which promotes gut modulation. Another pathogenic bacteria type is *Helicobacter pylori*, which resides in the oral cavity and stomach in patients with periodontitis. A study that followed patients for ten years shows how periodontal health and *H. pylori* can affect the prognosis of colon, gastric, and colorectal cancer patients. Due to the high prevalence of gastrointestinal (GI) cancer mortality among periodontitis patients, this study highlighted the association of GI mortality and periodontitis in cases of *H. pylori* infection, which determined that the infection has a certain risk of mortality and decreases survival outcomes [24].

**Figure 2:**
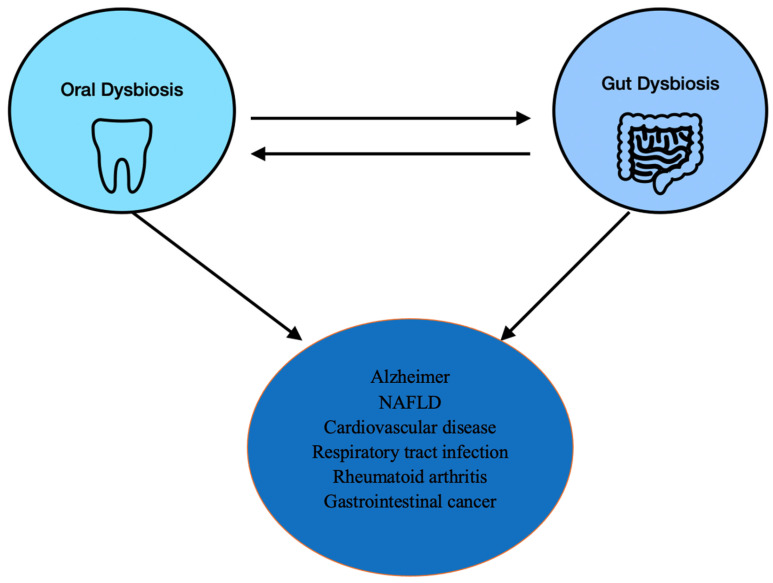
Bidirectional relationship of oral and gut dysbiosis. Effect on systemic diseases and gastrointestinal cancerous lesions [13,26].

A spread of periodontic pathogens via the circulatory system has already been found in humans and is associated with various systemic diseases, such as Alzheimer’s, cardiovascular disease, oral/colorectal cancer, gastrointestinal diseases, respiratory tract infections, bacterial pneumonia, adverse pregnancy outcomes, and diabetes [13,25]. All these consequences have been associated with oral cavity dysbiosis, which then alters the gut microbiota that affects the body systemically [26]. Gut dysbiosis can impact bile salt metabolism, weakening the body’s ability to combat inflammation, disrupting metabolic function, and being linked to diseases such as asthma, IBD, NAFLD, RA, hypertension, hepatitis, liver cancer, and obesity; these conditions, in turn, alter gut bacteria by causing dysbiosis, which is bidirectionally affected by toxins, proinflammatory factors, and pathogens, which are spread systemically by the circulatory system through the intestinal barrier, leading to the development of various inflammatory diseases in the body [13,15,26] (Figure 2).

#### 5.3.1. Esophagus and Stomach

Esophageal cancer (EC) is the sixth leading cause of cancer mortality; around 500,000 people die yearly from EC. The two main types of esophageal cancer are esophageal squamous cell carcinoma (ESCC) and esophageal adenocarcinoma (EAC). These two subtypes are histologically and clinically differentiated, with ESCC comprising 80% of EC cases [27].

A prospective study was performed that analyzed 199 cases with EAC. It has been shown that patients with periodontal disease have a 43% increased chance of developing EAC. In comparison, patients with a history of periodontitis and tooth loss have a 59% increased risk of developing EAC compared to patients with no history of periodontal disease and tooth loss [28]. Additionally, poor oral hygiene and alcohol consumption have been associated with a higher chance of developing ESCC [29].

Growing evidence shows that various periodontal pathogenic bacteria, such as *T. forsythia* and *P. gingivalis*, have been associated with the presence or risk of EC [28]. In cases of ESCC, *F. nucleatum* exists at high levels in cancerous tissue. Others, such as *P. gingivalis* and *Prevotella*, were also high in the tissue, while *Streptococcus* decreased in the samples [30]. The linkage between the microbiota and the pathogenesis of EC started growing before it was stated that *Streptococcus anginosus* is the leading cause of EC. Still, recent reports show that *Treponema denticola* and *S. mitis* infect the esophageal cancerous tissue. To sum up, *S. mitis* does not just infect the cancerous tissue; it is also one of the earliest colonizers of dental biofilm, which causes periodontitis. Meanwhile, *F. nucleatum* has been associated with the progression of ESCC, since it was found in patients with poor prognoses [31,32].

Gastric cancer is described as cancer in the stomach. Gastric cancer can arise due to infections such as *H. pylori*, which increases the risk of cancer in the lower and middle parts of the stomach. In contrast, gastroesophageal reflux disease (GERD) increases the risk of cancer in the upper stomach [33]. *H. pylori* is associated with periodontal disease but is also the primary etiology for various gastric diseases, such as chronic gastritis, GERD, and peptic ulcer. Oral *H. pylori* in the oral cavity is believed to be a resident pathogen that causes gastroesophageal infections. A study was conducted on 564 subjects with periodontal disease, of whom 278 had gastric complications such as gastritis and GERD. The study concluded that oral *H. pylori* can progress the severity of the gastric infection [34].

#### 5.3.2. Pancreas

The presence of circulating antibodies to selected oral pathogens due to periodontal disease has been associated with an increased risk of pancreatic cancer [26]. A study was conducted using mouthwash in 361 patients with pancreatic adenocarcinoma, where the presence of *P. gingivalis* and *A. actinomycetemcomitans*, along with Fusobacteria and *Leptorichia*, acted as a risk factor for pancreatic cancer. The presence of *P. gingivalis* increased cancer development by 59% in comparison with *A. actinomycetemcomitans*, which increased the risk by 50%. This evidence-based result shows us that periodontal pathogens can spread to the pancreas and contribute to the etiology of pancreatic cancer [35].

#### 5.3.3. Gallbladder 

Cholecystitis is described as the inflammation of the gallbladder, which can be due to gallstones or other complications [36]. A prospective study was carried out that involved around 65,869 postmenopausal women with periodontitis. Information was provided through questionnaires that were tracked for several years. The study concluded that postmenopausal women with a history of periodontitis had a 73% higher risk of developing gallbladder cancer compared to those women without a periodontal disease history [36].

Another retrospective study that involved 25,315 Taiwanese patients with cholecystitis was to investigate the effect of severe periodontitis and the incidence of dental scaling on patients before they were diagnosed with cholecystitis. Individuals in the age range of 50–70 with severe periodontitis had a 43.6% increased risk of acquiring calculous or acalculous cholecystitis [37]. 

#### 5.3.4. Colorectal Cancer 

Colorectal cancer (CRC) is a cancer of the colon and rectum. CRC cases in young adults are rising, and many researchers are trying to understand the cause. A periodontal pathogen, *F. nucleatum*, has been found in the gut in abundant amounts. It rarely exists in healthy guts, but it has been found in abundance in colorectal cancer patients. These bacteria are associated with cancer recurrence and poor prognoses [38].

A study was conducted to investigate the relationship between periodontal disease and CRC. The study comprised 348 CRC cases and 310 controls. The controls were correlated, and factors such as age, sex, BMI, education, and lifestyle were adjusted to obtain a concrete result. Individuals with a history of periodontal disease turned out to have a 1.45 times higher risk of developing CRC [39].

## 6. Therapeutic Innovations 

The most common approach to oral dysbiosis is to improve personal oral hygiene and root scaling in cases of periodontitis [40]. In deep pockets, it can be hard to reach all bacteria as periodontal pathogens may residue in soft periodontal tissues, so combining treatment with local antimicrobial agents such as chlorhexidine, amoxicillin, and metronidazole is recommended. Systemic antibiotics are only indicated in cases of periodontitis, grade C. A new approach uses probiotics, a bacteria strain that modulates an inflammatory response and produces hydrogen peroxide, positively impacting systemic cytokines and inflammatory markers. Probiotics such as lactobacilli and bifidobacteria are commonly used. One study has shown that *L. salivarius* as a freeze-dried tablet caused a reduction in *P. intermedia*, *P. gingivalis*, *T. forsythia*, *T. denticola*, and *A. actinomycetemcomitans* in patients with periodontitis [41].

Similar to gut dysbiosis, probiotics have been used in patients with IBD, where a combination of microbes and substrates, including *lactobacillus*, *bifidobacterium*, *Streptococcus*, insulin, and oligofructose provided good results, such as reduced symptoms and increased amounts of *Lactobacillus acidophilus* and *Bifidobacterium animalis* in stools after one week follow-up [19]. Many studies have shown a connection between periodontitis and IBS by administering Lactobacillus to treat gastrointestinal diseases and increasing β-defensin levels in mice’s intestinal and gingival tissues, decreasing inflammatory factors during periodontitis [42].

According to the Mendelian randomization test, since the oral cavity is the main opening for the body, gut microbiota can have some effect on oral microbiota. However, the mechanism is still unknown [22]. Still, we already know that oral microbiota can affect the gastrointestinal system [3]; this opened a gateway for researchers to diagnose systemic diseases by analyzing microbiota. Recently, an oral rinse has been developed to detect gastric cancer. The concept is built on studying the microbiome, which differs distinctly between individuals without gastric cancer and those who have acquired gastric cancer [43]. Since *H. pylori* infection is the leading cause of gastric cancer, new approaches are being taken due to the increasing resistance of bacteria. One is through treating periodontitis at the same time as curing gastric infection; this was a clinical trial, in which 98 patients with gastric *H. pylori* were enrolled. Forty-seven patients received triple therapy, which consisted of a proton pump inhibitor (PPI) with two antibiotics (clarithromycin and amoxicillin/metronidazole). The remaining 51 participants received triple therapy in addition to periodontal treatment. The results showed that triple therapy plus periodontal treatment had an eradication rate of 64.7% compared to triple therapy alone, which had an eradication rate of 51.5%. Periodontal treatment increased the success rate due to removing plaque, which consists of *H. pylori*, and is protected from systemic antibiotic therapy, which can act as a potential source of gastric reinfection [44].

The analysis of saliva microbiota is a promising diagnostic indicator of systemic diseases because it is easy to access and non-invasive. Changes in salivary microbiota contribute to the identification of biomarkers for many systemic conditions. The saliva microbiota test has been used to diagnose metabolic diseases, cardiovascular diseases, respiratory infections, RA, adverse pregnancy outcomes, IBD, Alzheimer’s disease, and autism spectrum disorders [45].

## 7. Discussion

Periodontal pathogens have been strongly linked to systemic diseases. The oral cavity harbors over 30 abundant species that produce endotoxins, which can hematogenously migrate and invade distant tissues [25]. *P. gingivalis* is a key pathogen that enters the cardiovascular system through epithelial ulcers and lymphatic vessels after periodontal treatment. This spread even contributes to atherosclerosis pathogenesis by inducing oxidative stress in endothelial cells, triggering an immunological response, promoting foam cell formation, and calcifying vascular smooth muscle cells. Ultimately, this exacerbates the atherosclerotic process [46].

However, the impact of periodontal pathogens extends beyond the cardiovascular system. The spread of microbial products into the circulatory system induces a systemic inflammatory state, linking periodontal pathogens to inflammatory diseases such as diabetes and RA [25]. Among the leakage of microbial products, the spread of inflammatory cytokines such Il-6 occurs; this is a cytokine that is connected to bone loss in periodontitis and which has also been linked to joint erosion in RA patients and other inflammatory diseases [47]. The spread of periodontal pathogens from the oral cavity can also occur through the external route by colonizing the gastrointestinal tract, serving as a reservoir for the development of gastrointestinal infections. These include periodontitis-associated IBD and gastric infections, such as those caused by *H. pylori*, which has been found in periodontal pockets and is believed to act as a reinfection source for gastroesophageal infections [3,34].

Periodontal pathogens have also been pointed out to be associated with neoplastic activity [11,30]. Notably, *P. gingivalis* has been found at high levels in esophageal and pancreatic specimens [28,30,35]. It has been shown that cancer occurs in the pancreas due to increased IL-6 production, which promotes the growth and invasion of oral squamous cell carcinoma. *P. gingivalis* interacts with protein citrullination and cytokine activity, modulating the immune response and driving tumor progression. Furthermore, it inhibits cell apoptosis by affecting cell cycle proteins such as p53, PI3K, and Bcl-2, leading to the proliferation and prolonged survival of cancerous tissue [48]. A similar mechanism has been observed in esophageal-induced tumor models, where *P. gingivalis* exhibits a tumorigenic effect by inducing inflammation through IL-6 interleukin secretion [30].

*F. nucleatum* has been detected in the esophagus and pancreas, and its presence in colorectal cancer patients has garnered significant attention, as it is typically absent in healthy colons [30,35,38]. Recently, NIH-funded research investigated *F. nucleatum* strains from the mouth could promote formation of colorectal tumors. Fna C2, a subspecies of *F. nucleatum*, was found in the colorectal cancerous tissue and mouth. Experiments in mice with colitis further confirmed that those infected with *Fna C2* developed more tumors compared to other subtypes. This discovery may inspire therapeutic drug design targeting this strain in the future [38].

Periodontal pathogens, such as *P. gingivalis*, *F. nucleatum*, and *T. forsythia*, have been frequently detected in esophageal, pancreatic, and colorectal tissues, where they are believed to promote carcinogenesis [28,30,35,38,49]. The underlying mechanisms, supported by in vitro and in vivo studies, involve chronic inflammation, immune system suppression, cell invasion and proliferation activation, anti-apoptotic activity, and carcinogenic substance production [30,48,49]. Although there is significant evidence linking these pathogens to cancer development, gaps remain in our understanding of their contributions due to limitations in in vitro and in vivo studies. Further research is needed to explore the signaling pathways promoted by these pathogens. Elucidating these mechanisms will enhance diagnostic, preventive, and therapeutic strategies, ultimately increasing treatment efficacy [49].

Dysbiosis caused by periodontitis has been shown to influence gut microbiota composition. An exploratory study which examined stool samples in patients with periodontitis demonstrated a reduction in alpha diversity in the gut microbiome. This dysbiotic shift is characterized by a decrease in beneficial bacteria and an increase in pathogenic species like *Proteobacteria* and *Fusobacteria*; here, large amounts of oral periodontal pathogens, such as *P. gingivalis*, *Fusobacterium*, *Tannerella*, and *Treponema*, have been detected in stool samples, which support the concept of the translocation of oral bacteria into the gut [18].

Periodontitis is linked to IBD through shared microbial and inflammatory pathways [13,21]. However, gaps in the literature prevent clear directionality and causality between periodontitis and IBD. Since IBD is a multifactorial disease, oral and gut dysbiosis alone may not predispose individuals to periodontitis-associated IBD. Instead, the influx of oral pathogens combined with impaired gut resistance promotes the condition. Confounding risk factors in existing cross-sectional studies, such as lifestyle and tobacco use, limit our understanding of the causal relationship and whether periodontitis occurs before, after, or simultaneously with IBD [21]. Although studies on induced colitis in mice and Yamazaki’s demonstration of oral pathogens ectopically colonizing the gut provide valuable insights, their limitations should be considered separately when exploring the IBD–periodontitis interconnection. Longitudinal studies are needed to reduce confounding factors and establish a better understanding of the IBD–periodontitis relationship [3,21].

Despite the gaps and limitations in the current research, the evidence still shows that periodontal pathogens can alter the gut microbiota, leading to dysbiosis [3]. Until now, there has been limited evidence for the potential improvements that IBD treatment can bring for the state of periodontal disease and vice versa. Periodontal therapy has been shown to modulate gut microbiota by lowering harmful bacteria in both experimental and clinical trials; on the other hand, IBD treatments have been linked to better oral defense mechanisms and improved periodontal healing, which indicates a shared microbial and inflammatory pathway of the diseases [21].

In addition to the impact of periodontal treatment on the gut, it has shown benefits in other gastrointestinal diseases such as gastric infection caused by *H. pylori*. In a clinical trial, patients who received both periodontal treatment and standard *H. pylori* therapy acquired significantly higher eradication rates compared to those receiving only standard therapy, which underscores the importance of combining oral health management in treatments [44]. Growing evidence of how periodontal pathogens impact overall health directly and indirectly emphasizes the integration of the oral cavity into treating and diagnosing systemic diseases [13,25,48]. Consequently, oral microbiota analyses and oral rinses have gained attention as diagnostic tools for detecting a range of systemic diseases, such as gastric cancer, metabolic diseases, cardiovascular diseases, respiratory infections, RA, adverse pregnancy outcomes, IBD, and neurodegenerative diseases [25,43,45].

These studies and research findings demonstrate the pathological role of periodontal bacteria in promoting gastrointestinal cancer, while highlighting the bidirectional relationship between oral and gut bacteria, where treating one aspect can benefit the other. Additionally, these studies and diagnostic tools are gaining attention, inspiring future innovations and research focused on controlling gut microflora and halting periodontitis progression; this highlights the importance of periodontal treatment in the management and diagnosis of systemic diseases at early stages [19]. Although further work is needed to address the existing gaps and to gain a deeper understanding of the pathological interactions of periodontal bacteria that promote systemic diseases, future research holds promise. Advancing our knowledge will enhance diagnostic, preventive, and therapeutic strategies, ultimately increasing treatment efficacy [21,49].

## 8. Conclusions

The dysbiotic change in the oral cavity due to periodontitis is linked directly and indirectly to systemic diseases such as IBS, neurodegenerative diseases, muscle joint diseases, respiratory infections, and gastrointestinal cancer; this underscores the importance of maintaining oral hygiene for the prophylaxis of oral diseases and the prevention of systemic diseases. A better understanding of the interconnections between oral health and systemic diseases will integrate oral health management to offer new prevention, diagnostic, and treatment opportunities, improving overall patient outcomes.

## 9. Recommendation for Future Research

This research provides a solid foundation for understanding the link between periodontitis and systemic diseases, shedding light on the underlying pathogeneses and mechanisms. To further advance our knowledge, we recommend the following areas of focus:

- Develop treatments that target both oral and gut dysbiosis, which could help reduce periodontal disease progression and improve gut health.
- Conduct randomized clinical trials to investigate the periodontitis–IBD connection and optimize treatment approaches.
- Investigate therapeutic drugs that target Fna C2 strains in colorectal cancer.
- Explore how gut dysbiosis influences periodontitis, as this mechanism is not yet fully understood.
- Examine how periodontal pathogens promote various signaling pathways in carcinogenesis, which could reveal opportunities for prevention and targeted interventions.

## Data Availability

No new data were created.
